# Quality of reporting on randomized controlled trials on recurrent spontaneous abortion in China

**DOI:** 10.1186/s13063-015-0665-6

**Published:** 2015-04-18

**Authors:** Jie Gao, Gaopi Deng, Yunyun Hu, Yanxi Huang, Liming Lu, Dandan Huang, Yadi Li, Lin Zhu, Xiaojing Liu, Xin Jin, Songping Luo

**Affiliations:** Department of Gynecology, First Affiliated Hospital, Guangzhou University of Chinese Medicine, Guangzhou, 510405 China; Guangdong Provincial Hospital of Chinese Medicine, Guangzhou, 510120 Guangdong China; Department of Anorectal, First Affiliated Hospital, Guangzhou University of Chinese Medicine, Guangzhou, 510405 China

**Keywords:** CONSORT statement, Recurrent spontaneous abortion, Quality of reporting, Randomized controlled trial

## Abstract

**Background:**

Despite increasing numbers of RCTs done in China, detailed information on the quality of Chinese RCTs is still missing. The aim of this study was to assess the reporting quality of RSA RCTs and to identify significant predictors of reporting quality.

**Methods:**

A literature review was conducted with the aim of identifying published RCTs on RSA conducted in China. In order to rate the report quality, we scored 1 for the item of CONSORT 2010 if it was reported and 0 if it was not stated or unclear. An overall quality score (OQS) with a range of 0–15 and a key methodological index score (MIS) with a range of 0–3 were calculated for each trial.

**Results:**

A total of 98 relevant RCTs were included in the final analysis. The median OQS was 7, with a minimum of 1 and maximum of 12. The general level of OQS was not high, especially among ‘sample size,’ ‘baseline data,’ ‘outcomes and estimation,’ and ‘ancillary analyses,’ all of which had a positive rate of less than 10%. The median MIS was 1 with a minimum of 0 and maximum of 1. ‘Allocation concealment,’ ‘blinding,’ and ‘intention-to-treat analysis’ were mentioned in 1 (1%), 1 (1%) and 69 (70%) of the studies, respectively. In univariate analysis, funding was the only factor associated with an increased OQS. Specifically, the mean OQS increased by approximately 1.52 for manuscripts supported by funding (95% *CI*: 0.12 – 2.92; *p* = 0.03). With regard to the MIS, no association was found for any variable.

**Conclusion:**

RCTs of RSA conducted in China need improvement in order to meet the level of “reporting quality” required by the CONSORT statement.

**Electronic supplementary material:**

The online version of this article (doi:10.1186/s13063-015-0665-6) contains supplementary material, which is available to authorized users.

## Background

Recurrent spontaneous abortion (RSA) is the occurrence of three or more consecutive pregnancies that end in miscarriage of the fetus before viability [1]. The estimated frequencies of three or more and two or more consecutive pregnancy losses are 0.9% and 4.2%, respectively [2]. RSA affects 1% to 5% of women of reproductive age in China [3] and is one of the most difficult forms of infertility affecting Chinese women, thus being a pressing issue [4]. RSA can both physically and psychologically harm patients, and additionally bring heavy economic burdens upon families. Hence, research on the prevention and treatment of recurrent miscarriage is of significance both clinically and socially. Since the etiology and pathogenesis of RSA are largely unclear, various forms of intervention have been used in clinical practice, but the majority of them still lack sufficient evidence to show that they do prevent miscarriages in women with RSA [5].

To find the most efficient treatment, more and more randomized controlled trials (RCTs) in RSA have been appearing [6-8]. RCTs, as the ‘gold standard’ of evidence-based clinical practice, are generally considered to have the highest level of credibility in determining the efficacy of a new treatment [9]. Moreover, the quality of reporting is essential for guiding journal peer-review decisions, for experts’ recommendations and for conducting unbiased meta analyses as it influences the interpretation of evidence. Understanding the importance of transparency in reporting clinical trials, an international team, including epidemiologists, statisticians and journal editors, developed the Consolidated Standards for Reporting Trials (CONSORT) Statement in 1996. Since then, more and more researchers have been using CONSORT to assess reporting quality [10-14].

Nevertheless, to the best of our knowledge, there has been no recent special report on the quality of RCTs of RSA conducted in China. The aim of this study is to assess the overall reporting quality of published articles of randomized trials of RSA with a special focus on the key methodological items that safeguard against biases, namely concealment of allocation, appropriate blinding, and analysis according to the intention-to-treat principle. Second, we also aimed to identify significant predictors of reporting quality.

## Methods

### Search strategy

A systematic and comprehensive literature review was conducted with the aim of identifying published RCTs of RSA conducted in China. We searched the following bibliographic databases: Embase (1980 to May 2014), Medline (1966 to May 2014), CINAHL (1982 to May 2014), China National Knowledge Infrastructure (CNKI, 1979 to May 2014), the Chinese Biological Medicine Database (CBM-disc, 1979 to May 2014), Wanfang databases (1982 to May 2014) and VIP database (1992 to May 2014). In addition, we searched the Cochrane Central Register of Controlled Trials (CENTRAL) (May 2014). Additionally, reference lists of eligible studies and previous systematic reviews were also reviewed to identify further eligible studies. The search terms used were in Chinese and English and included keywords such as spontaneous abortion, recurrent abortion, fetal loss, miscarriage, randomized trials, RCT and China.

### Inclusion and exclusion criteria

Types of studies: We looked at randomized controlled trials that assessed the effect of a particular treatment in women with recurrent miscarriages. However, quasi-randomized trials, nonrandomized, cross-over RCTs, case reports and case-control studies were excluded. All RCTs had to be performed in China and primarily by Chinese researchers.

Participants’ criteria: We included pregnant women with a history of three or more consecutive unexplained miscarriages prior to 24 weeks of gestation. The target population of this review was women with miscarriages that remained unexplained after routine investigations.

### Assessment of reporting quality

#### Overall

Given that we defined quality of reporting as the extent to which the rationale, method, conduct and results of the trial were reported, we adopted 15 relevant items from the revised CONSORT statement for our appraisal. CONSORT items were chosen because a lack of their reporting has been associated with estimates of increased levels of bias [15,16]. An overall quality score (OQS) with 15 items from the revised CONSORT statement was used as previously described [16-18]. We scored 1 for an item of CONSORT 2010 if it was reported, and 0 for an item if it was not stated or unclear. For the overall quality score (OQS), 15 items were scored and calculated with a range of 0–15 (Table 1) [17-19].

**Table 1:** **Overall quality of reporting rating using items from the CONSORT Statement (** ***n*** **= 98)**

**Item**	**Criteria**	**Description**	**No. of positive trials**	**%**	**Cohen’s** ***к*** **coefficient**	**95% CI**
1	“Randomized” in the title or abstract	Study identified as a randomized controlled in the title or abstract	10	10	1.00	-
2	Background	Adequate description of the scientific background and explanation of rationale	85	87	0.67	0.43 to 0.91
3	Trial design	Description of trial design (such as parallel, factorial) including allocation ratio	84	86	0.78	0.54 to 0.96
4	Participants	Description of the eligibility criteria for participants	72	74	0.72	0.49 to 0.93
5	Interventions	Details of the interventions intended for each group	80	82	0.62	0.41 to 0.95
6	Outcomes	Definition of primary (and secondary when appropriate) outcome measures	82	84	0.75	0.48 to 0.97
7	Sample size	Description of sample size calculation	0	0	0.81	0.52 to 0.99
8	Randomization	Description of the method used to generate the random sequence	14	14	0.83	0.63 to 0.97
12	Statistical methods	Description of the statistical methods used to compare groups for primary outcomes, subgroup analyses or adjusted analyses	71	72	1.00	-
13	Flow chart	Details on the flow of participants through each stage of the trials (no. of patients randomly assigned, receiving intended treatment, completing the protocol and analyzed)	87	88	0.77	0.61 to 0.97
14	Recruitment	Dates defining the periods of recruitment and follow-up	79	81	0.73	0.42 to 0.98
15	Baseline data	An outline of baseline demographic and clinical characteristics of each group	3	3	0.68	0.35 to 0.92
17	Outcomes and estimation	For each primary and secondary outcome, a summary of results for each group is given, along with the estimated effect size and its precision (e.g., 95% CI)	9	9	0.72	0.43 to 0.96
18	Ancillary analyses	Clear statement of whether subgroup/adjusted analyses were pre-specified or exploratory	8	8	0.74	0.37 to 0.95
19	Harms	Description of all important adverse events in each group	12	12	0.82	0.69 to 0.99

#### Key methodological items

Three key methodological categories of allocation concealment, blinding and intention-to-treat (ITT) analysis have been assessed separately because they relate to potential sources of bias [20-22]. We then developed eight “yes”/“no” items (Table 2) and wording that emphasized quality of reporting rather than adequacy of trial design. Each item was scored 1 if the method was appropriate and 0 if it was inappropriate or if the reporting was unclear.

**Table 2:** **Reporting quality of key methodological items (** ***n*** **= 98)**

**Item**	**Criteria**	**Description**	**No. of positive trials**	**%**	**Cohen’s** ***к*** **coefficient**	**95% CI**
9	Allocation concealment	Description of the method used to implement the random allocation sequence assuring the concealment until interventions are assigned	1	1	1.00	-
11	Blinding	Whether or not participants, those administering the interventions or those assessing the outcomes were blinded to group assignment	1	1	0.68	0.50 to 0.96
16	Intention-to-treat analysis	No. of participants in each group included in each analysis and whether it was done by “intention to treat”	69	70	0.72	0.45 to 0.98

#### Data extraction

Each article was reviewed by two independent investigators (Yunyun Hu and Yanxi Liu) who had received training in research methodology and statistics using modified CONSORT checklists. In addition the two investigators were blinded to each other’s ratings and completed the rating form independently. Information was extracted as the modified CONSORT checklist with 18 items (Tables 1 and 2). We judged the consistency of two assessors by calculating Cohen’s *к*-statistic. The consistency judgment criteria were as follows: if *к =* 1, agreement was judged as “perfect;” if *к* was between 0.8 and 1, agreement was “good;” if *к* was between 0.6 and 0.8, agreement was “substantial;” if *к* was between 0.4 and 0.6, agreement was “moderate;” if *к* was between 0.2 and 0.4, agreement was “fair;” if *к* was less than 0.2, agreement was “poor” [17,18].

### Data analysis

The characteristics of the publications OQS and MIS were then described by descriptive analysis. To screen factors associated with OQS, it was used as the outcome variable and the characteristics of the publications as independent variables, which were modeled using linear regression. A multivariate regression model was performed only if variables in the univariate models were significant at *p* ≤ 0.10. In the final multivariate model, variables were significant predictors at *p* ≤ 0.05. As the outcome variable, MIS could be considered as a dichotomy, and the logistic regression model was used to select factors associated with MIS. Descriptive statistical analysis as well as linear and logistic regression analysis was performed using SPSS version 20.0 (SPSS, Chicago, IL, USA). Analysis of Cohen’s k-statistics was performed using SAS software, version 9.3 (SAS Institute, Inc., Cary, NC, USA). The database of RCTs of RSA conducted in China are provided in Additional file 1.

## Results

**Figure 1:**
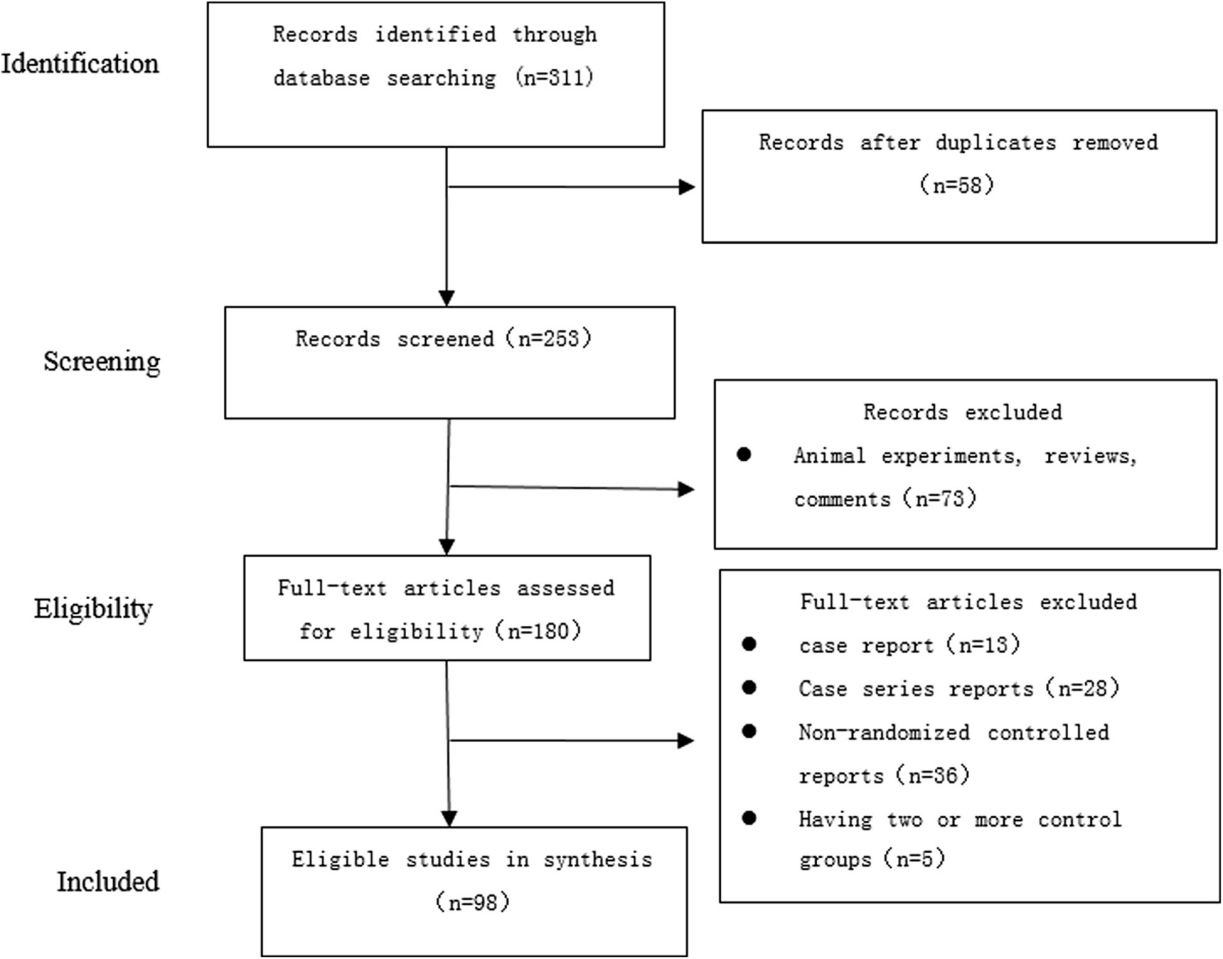
The article selection process.

The RCTs selection process is outlined in Figure 1. The researchers applied the search method to find 311 reports related to the topic. After careful selection, a total of 98 relevant RCTs were included in the final analysis (see Figure 1).

### Characteristics of included trials

There has been a spurt of growth based on the 76 (77.6%) from the years 2009–2014 compared with 22 (22.4%) from 1998–2008. Twenty-three articles (23.5%) were related to modern medicine, especially immunotherapy; 42 (42.9%) were related to Chinese medicine, and the remaining 33 (33.7%) combined both methods. As for source of finance, eight articles (8.2%) reported the funding sources, half of which were obtained from provincial sources, while the rest were from municipal sources. All cases were from the same hospital in each paper. Finally, in terms of choice of comparator interventions, the largest number of interventions comprised intramuscular injection of progesterone followed by an intramuscular injection of human chorionic gonadotropin (HCG).

Rater agreement: Substantial agreement was observed for items 2, 3, 4, 5, 6, 11, 13, 14, 15, 16, 17 and 18; good agreement was observed for items 7, 8 and 19; perfect agreement was observed for items 1, 9 and 12 (see Tables 1 and 2).

### Quality of reporting

#### Overall

The ratings of overall reporting quality are listed in Table 1. The level of reporting was below average as the mean OQS was 7.10 with a standard deviation of 1.95. According to our research, part of the information was insufficient or inadequate in most studies. The general level of reporting was not high, especially for the items ‘sample size,’ ‘baseline data,’ ‘outcomes and estimation’ and ‘ancillary analyses,’ each with a positive rating of less than 10%. The lowest ratings were for ‘randomization’ and ‘harms,’ both with a positive rating of less than 50%. However, other items such as ‘background,’ ‘trial design,’ ‘interventions,’ ‘outcomes’ and ‘flow chart’ each received a high rating of over 80%.

#### Key methodological items

‘Allocation concealment,’ ‘blinding’ and ‘intention-to-treat analysis’ were mentioned in 1 (1%), 1 (1%) and 69 (70%) studies, respectively. The median MIS was 1 with quartile interval of 1. And 29 papers (29.6%) did even not mention either of them.

#### Exploratory analysis: factors associated with better reporting quality

Based on the multicollinearity diagnostics, there was no statistical correlation between different explanatory variables. In univariate analysis, funding was the only factor associated with an increased OQS. As only one variable was included in univariate analysis, the multivariate linear regression model was presented with only one explanatory variable. The multivariate linear regression model was built successfully in statistics (*F* = 4.64, *p* = 0.03). Specifically, the mean OQS increased by about 1.52 for manuscripts supported by funds (95% *CI*: 0.12–2.92; *p* = 0.03).

With regard to the MIS, in univariate logistic regression analyses, no variable could be included in this model, and the following multivariate logistic regression model was not performed Table 3.

**Table 3:** **Multivariate linear regression analysis for factors associated with better overall quality of reporting rating using items from the CONSORT statement (** ***n*** **= 98)**

**Variables**	***β***	**SE**	***t***	***P***	**95% CI**
Funding	1.52	0.71	2.16	0.03	0.12 – 2.92

## Discussion

This study has demonstrated that the qualities of reporting were not met from 1998 to 2014. This result suggests that RCTs of RSA conducted in China need to improve in order to meet the level of “reporting quality” required by the CONSORT statement. Low-scored items were related to the Methods and Results sections as follows: ‘ancillary analyses,’ ‘allocation concealment,” randomization,” sample size,’ ‘blinding,’ ’baseline data’ and ‘analysis by ITT.’ These key methodological categories, which are regarded as being highly correlated to avoid bias, emerged as the worst.

Well-designed and implemented randomized controlled trials (RCTs) have the strongest power to prove the efficiency of interventions. Randomization is now considered the best method to ensure that the baselines between groups are similar and to avoid removing selection bias between them. According to our study, only 14 articles (14.3%) reported the correct methods to obtain the allocation sequence by a random number table or computerized random number generator. Others created inaccuracies by dividing groups using birth date or admission number, which is regarded as not choosing individuals correctly because of the inability to conceal these allocation systems adequately.

Most articles included in our research did not adequately consider allocation concealment as the positive rate in our research was only 1% (1/89). This was below 30-50% in other evaluation reports related to trials in obstetrics and gynecology [23-25]. We recommend using an allocation concealment that can prevent foreknowledge of treatment and protect the enrolled participants from being influenced. By using this method, we could implement a generated allocation schedule [26]. A lack of allocation concealment may compromise the unpredictable allocation sequence and cause selection bias, reducing the strength of conclusions. Compared with trials that reported allocation sequences adequately, the inadequately or unclearly concealed ones might yield larger estimates of treatment effects. Blinding is another important safeguard to ensure the quality of articles against performance and ascertainment bias, especially when assessing subjective outcomes [25]. Although blinding is not always available to all trial participants, it is feasible to the patients and investigators in data collection and processing. Nonetheless, the majority of the 98 trials did not mention it: only one trial reported a description of blinding.

Sample size calculations need to strike a balance between statistical considerations and treatment effect difference compared to standard therapies. The author needs to show how the sample size was determined, as it not always easy to recruit enough participants within a set time besides those that withdraw. Approximately 66% failed to achieve their planned sample size based on a review of 41 RCTs [27]. No trial had clearly described the process of sample size calculation, and more than 30% (34/98) had a sample size less than 50. This result was poor when compared to 37.8% in the spine area [28]. We should pay more attention to sample size calculation as it indicates the difference between new interventions and controlled ones. Furthermore, very small-scale RCTs are often insufficient to evaluate benefits and more likely to cause bias risks.

For the reporting status of RCTs of RSA conducted in other countries, David et al. [6] found approximately 14% of RCTs reported items of random sequence or allocation concealment, and more than 50% of RCTs described the blinding method in the field of progestogen for preventing RSA. Morley et al. [7] found that 40% of RCTs reported items of random sequence or allocation concealment, and 60% described the blinding method in the field of human chorionic gonadotrophin (hCG) for preventing RSA. From the comparison between the status of RCTs of RSA conducted in China and other countries, it seems that the situation in China is worse according to these key methodology items.

Our analysis has revealed that research supported by funding was significantly associated with better OQS based on the CONSORT statement. Compared with the funding resource rate, which was 8% (8/98) in this study, it was below 56% for Hodgkin’s lymphoma in biomedical journals [29] and 66% for epidemiology [30]. Since large-size trials cost more, some RCTs with smaller sample size than necessary were performed in case of lack of sponsorships, which typically led to negative conclusions.

Several suggestions could be made considering our results, which could enhance the reporting quality of future RCTs of RSA. First, several items, such as ‘ancillary analyses,’ ‘allocation concealment,’ ’ randomization,’ ‘sample size,’ ‘blinding,’ ‘baseline data’ and ‘analysis by ITT’ should be paid more attention when RCT reports are prepared. Clinical research training and consultation with statisticians and epidemiologists are also important in order to improve report quality. Second, funding applications should be encouraged as they can guarantee resources for RCT design, execution and evaluation. This study does have its limitations. First, each article enrolled was published in Chinese. There were no unpublished or currently in progress articles included, which may lead to publication bias. Second, to evaluate the quality of reporting in RCTs quantitatively, according to some rating methods published in previous studies [17-19], we extracted major items, not all items, from the CONSORT 2010 Statement. Third, satisfying some Consort recommendation in the report did not imply that the trial really fulfilled it. For example, a report may state that the analysis was by ITT, but in actuality several randomized patients were excluded from the report. Despite these limitations, we think our results have good internal validity as agreement was judged substantial, good or perfect in the evaluation process executed by two independent assessors.

## Conclusion

Our findings show that the reporting quality of RCTs in recurrent miscarriage is unsatisfactory. Regarding the crucial methodological issues of allocation concealment, blinding and sample size calculation, our results stress the need to improve the reporting quality of RCTs of RSA conducted in China.

## Additional file

Additional file 1:
**CONSORT evaluation of RCT reports of RSA conducted in China.**

## Abbreviations

RCTs: Randomized controlled trials
RSA: Recurrent spontaneous abortion
OQS: Overall quality score
MIS: Methodological index score
CONSORT: Consolidated Standards for Reporting Trials
CNKI: China National Knowledge Infrastructure
CBM-disc: Chinese Biological Medicine Database
CENTRAL: Cochrane Central Register of Controlled Trials
ITT: Intention to treat
HCG: Human chorionic gonadotropin
